# Malignancy-associated metabolic profiling of human glioma cell lines using ^1^H NMR spectroscopy

**DOI:** 10.1186/1476-4598-13-197

**Published:** 2014-08-27

**Authors:** Wei Shao, Jinping Gu, Caihua Huang, Dan Liu, Huiying Huang, Zicheng Huang, Zhen Lin, Wensheng Yang, Kun Liu, Donghai Lin, Tianhai Ji

**Affiliations:** Chenggong Hospital and College of Chemistry and Chemical Engineering, Xiamen University, Xiamen, 361005 China; Research Institute of Exercise and Rehabilitation, Fujian Medical University, Fuzhou, 351009 China; School of Life Sciences, Xiamen University, Xiamen, 361005 China

**Keywords:** Glioma cell line, Malignancy, Metabolic profiling, ^1^H-NMR, Spectroscopy

## Abstract

**Background:**

Ambiguity in malignant transformation of glioma has made prognostic diagnosis very challenging. Tumor malignant transformation is closely correlated with specific alterations of the metabolic profile. Exploration of the underlying metabolic alterations in glioma cells of different malignant degree is therefore vital to develop metabolic biomarkers for prognosis monitoring.

**Methods:**

We conducted ^1^H nuclear magnetic resonance (NMR)-based metabolic analysis on cell lines (CHG5, SHG44, U87, U118, U251) developed from gliomas of different malignant grades (WHO II and WHO IV). Several methods were applied to analyze the ^1^H-NMR spectral data of polar extracts of cell lines and to identify characteristic metabolites, including principal component analysis (PCA), partial least squares discriminant analysis (PLS-DA), fuzzy c-means clustering (FCM) analysis and orthogonal projection to latent structure with discriminant analysis (OPLS-DA). The expression analyses of glial fibrillary acidic protein (GFAP) and matrix metal proteinases (MMP-9) were used to assess malignant behaviors of cell lines. GeneGo pathway analysis was used to associate characteristic metabolites with malignant behavior protein markers GFAP and MMP-9.

**Results:**

Stable and distinct metabolic profiles of the five cell lines were obtained. The metabolic profiles of the low malignancy grade group (CHG5, SHG44) were clearly distinguished from those of the high malignancy grade group (U87, U118, U251). Seventeen characteristic metabolites were identified that could distinguish the metabolic profiles of the two groups, nine of which were mapped to processes related to GFAP and MMP-9. Furthermore, the results from both quantitative comparison and metabolic correlation analysis indicated that the significantly altered metabolites were primarily involved in perturbation of metabolic pathways of tricarboxylic acid (TCA) cycle anaplerotic flux, amino acid metabolism, anti-oxidant mechanism and choline metabolism, which could be correlated with the changes in the glioma cells’ malignant behaviors.

**Conclusions:**

Our results reveal the metabolic heterogeneity of glioma cell lines with different degrees of malignancy. The obtained metabolic profiles and characteristic metabolites are closely associated with the malignant features of glioma cells, which may lay the basis for both determining the molecular mechanisms underlying glioma malignant transformation and exploiting non-invasive biomarkers for prognosis monitoring.

**Electronic supplementary material:**

The online version of this article (doi:10.1186/1476-4598-13-197) contains supplementary material, which is available to authorized users.

## Introduction

Gliomas, which are the most aggressive type of brain tumors, show high morbidity, a high recurrence rate, and high mortality. Survival from gliomas depends on the tumor type and grades of malignancy [1]. According to the World Health Organization (WHO) standards, gliomas are classified into four malignant grades. WHO I–II gliomas can be treated with surgery and chemoradiotherapy, and are generally associated with a survival time of 5 to 10 years. WHO III-IV gliomas have a survival time of only 9–12 months, because of the inefficacy of surgery and chemoradiotherapy. In addition, over 50% of low-grade gliomas undergo malignant transformation into high-grade gliomas within 5–10 years during recurrence [2, 3]. Malignant transformation of a glioma is a very complex process, which is associated with poor prognosis and reduced survival times. Hence, there are ongoing efforts to increase the understanding of glioma malignant progression.

To date, oncology research has shown that the malignant transformation of a tumor is closely related to cellular metabolism, mainly through the large-scale genetic and protein analyses [4, 5]. Nevertheless, variations in these upstream events are not sufficient to establish the molecular mechanisms of cell metabolic changes in glioma malignant progression. Metabolites, downstream of both transcription and translation, are potentially a better indicator of enzyme activity [6]. Thus, highthroughput metabonomics analysis is very helpful to gain a better understanding of the molecular mechanisms of glioma malignant transformation and to exploit biomarkers for prognosis monitoring. In the previous work, MacKinnon *et al.* performed metabolic profiling of glioma tissues and their normal counterparts [7]. Recent studies have further highlighted metabolic profiling of glioma tissues of different grades [8–10]. Despite considerable progress in understanding metabolomics profiling of glioma tissues, ischemia and hypoxia associated with tissue sample preparation might cause metabolic degradation, directly affecting the concentrations of specific metabolites between the low- and high-grade glioma [9]. As a prerequisite to the analysis of more complicated tumor tissues, cell lines are the most relevant model systems for exploring metabolic information that correlates with their biological characteristics. Moreover, data generated by metabolic profiling of individual cells could be controllable, highly stable, and repeatable [11, 12]. Despite these advantages, studies of cell lines are less extensive than metabolic analysis of glioma tissues.

In the present study, we conducted ^1^H NMR-based metabolic profiling of cell lines from glioma tissues of different malignant grades, focusing on metabolic profiles that are relevant to the malignant features. The malignancy-associated metabolites identified directly from cell lines’ spectra could be useful to further determine the molecular mechanisms underlying malignant transformation, and to provide a rational basis for developing non-invasive biomarkers for gliomas prognosis monitoring.

## Results

### Metabolic profiles analysis of glioma cell lines

**Figure 1:**
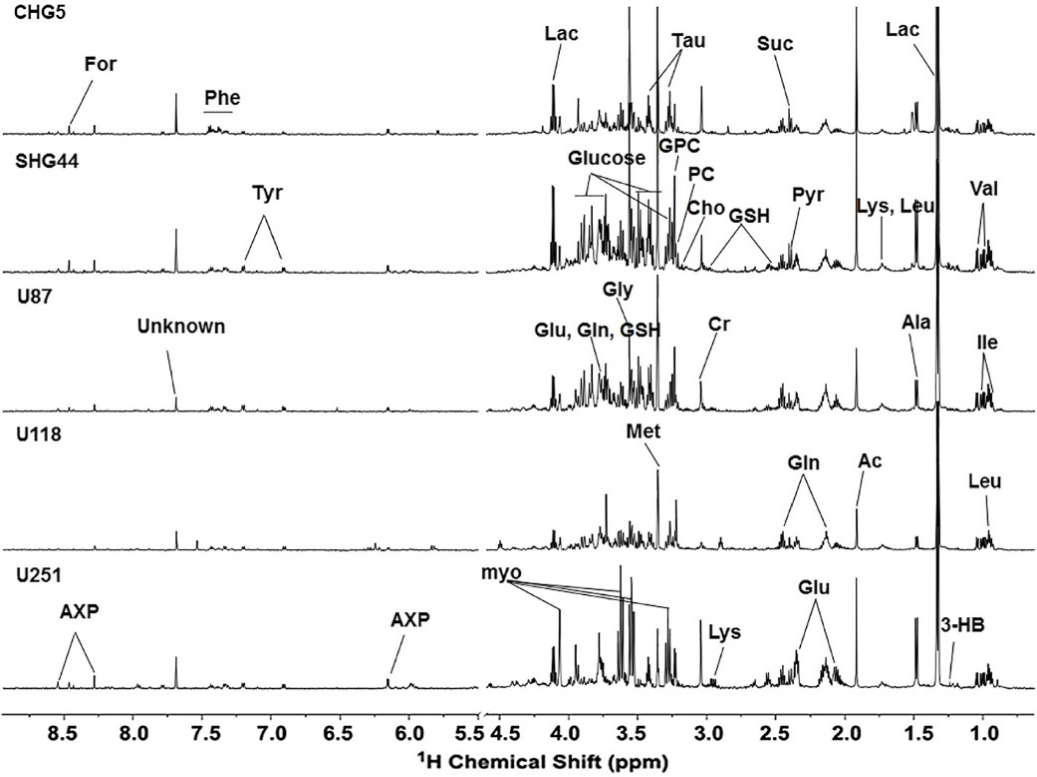
^**1**^ **H NMR spectra of aqueous metabolites from five glioma cell lines.** The spectral regions from 1.0 to 4.5 ppm and 5.5 to 8.5 ppm are shown. The water region (4.5–5.5 ppm ) was removed.

The five cell lines used in this study were derived from glioma tissues of different malignant degrees (shown in Table 1). Aqueous phase extracts of these cell lines were subjected to NMR analysis. The NMR spectra acquired 48 hours after seeding are shown in Figure 1. As the ^1^H NMR spectra from six or more measurements for each cell line displayed almost identical spectral profiles, only one of the replicates is shown in the Figure 1. These observations indicated that metabolic profiles of the independently grown cell lines were highly reproducible under the same culture conditions. The resonance assignments of major metabolites in the spectra were performed based on both literature data [13–15] and the Human Metabolome Database ( http://www.hmdb.ca). As a result, more than 30 metabolites were identified, which provided adequate information for assessing variations in metabolic profiles within the five cell lines.

**Table 1:** **Clinical sources of the glioma cell lines used in this study**

Designation	Source (Tumor stage)	P53 gene mutation
CHG5	WHO II	Unknown
SHG44	WHO II	Wild-type [16]
U87	WHO IV	Wild-type [17]
U118	WHO IV	Mutation [18]
U251	WHO IV	Mutation [16]

**Figure 2:**
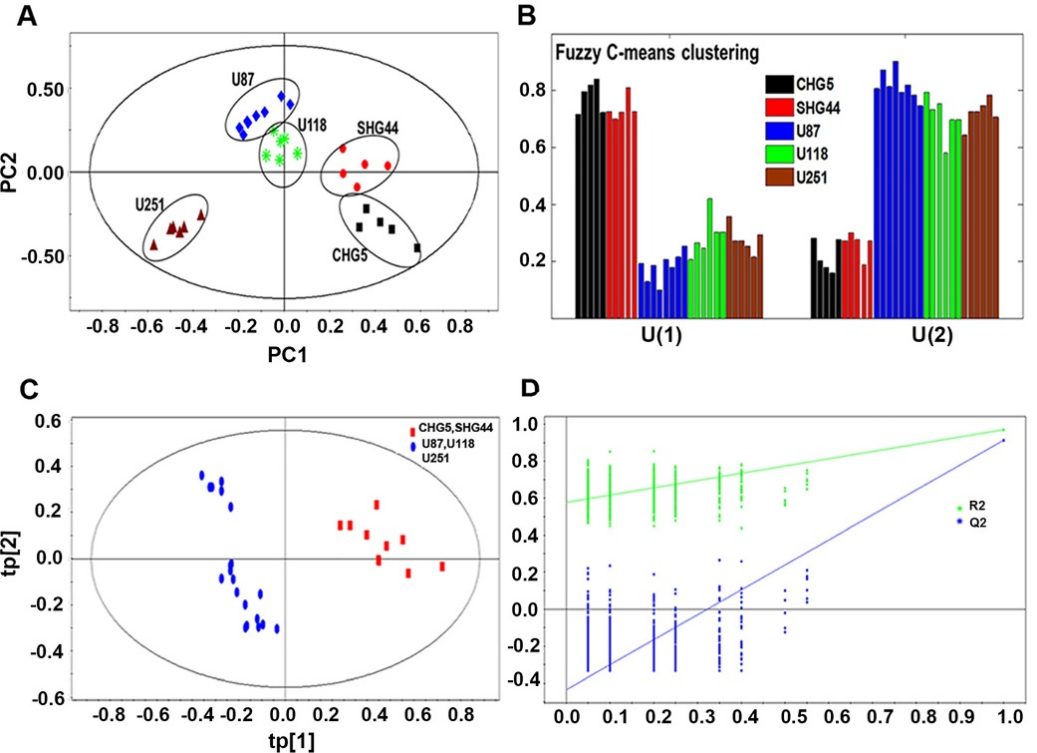
**Metabolic profiles of five glioma cell lines. (A)** PCA scores plot for spectral data of aqueous metabolites from five glioma cell lines. Each data point in ellipse represents a sample with replicates for each cell line. **(B)** FCM clustering for spectral data from five glioma cell lines. The cluster number was set at two and the membership values for each cell line were determined. **(C)** PLS-DA scores plot of the classification model. **(D)** The validation plot of the PLS-DA model, generated from the permutation test that was randomly permuted 999 times with the first three components. The green square is R2Y (cum), which is the explained variance of the model. The blue square is Q2 (cum), standing for the predictive ability of the model.

To determine the differences in the metabolic profiles among the five cell lines, we performed principal component analysis (PCA) to analyze the normalized ^1^H NMR data. The results showed that all samples were located in the Hotelling’s T2 oval of the 95% confidence interval, and the cell extracts from the same cell line clustered together (Figure 2A). Moreover, cell lines (CHG5, SHG44) from low-grade glioma were separated from those derived from high-grade glioma (U118, U87 and U251) along the first principal component (PC1).

To confirm the separation of groups from PCA, we utilized the fuzzy c-means clustering (FCM) method to analysis metabolic profile clustering by calculating memberships of each feature to each of the user-defined numbers of clusters. In this method, a membership value near 1 indicates strong membership and close to 0 indicates weak or no membership. The PCA scores plot (Figure 2A) provided visual information on the distribution of metabolic profiles, which suggested the number of clusters could be set to two. When the cluster number was two, CHG5 and SHG44 were grouped into one cluster whereas U118, U87 and U251 were grouped together into another cluster (Figure 2B). We further used projection to latent structure with discriminant analysis (PLS-DA) to evaluate the reliability of the two-cluster model and obtained three predictive principal components using the discrimination calculation. We then performed permutation tests with 999 iterations to assess the possibility of model over fitting. The obtained parameters were R2X (cum) = 0.560, R2Y (cum) = 0.964, Q2 (cum) = 0.933, which indicated that the model was highly reliable (Figure 2C and 2D). These results indicated that the two-cluster model possessed high discrimination and predictive capability. Notably, the result from the two-cluster model was fully consistent with the grouping of cell lines based on the pathological grade of the corresponding glioma tissues.

### The comparison of malignant behaviors of glioma cell lines

**Figure 3:**
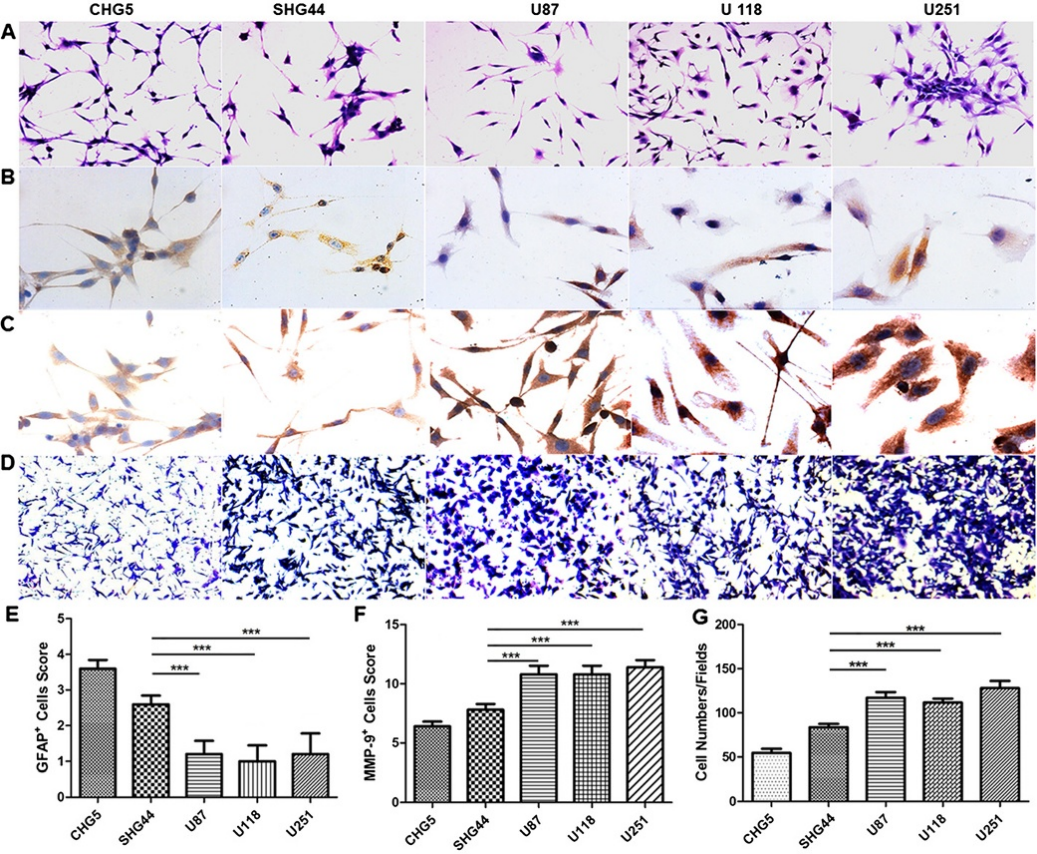
**Malignant features of five glioma cell lines. (A)** H&E staining of the five glioma cell lines. **(B)** Representative images of GFAP expression in glioma cell lines by IHC staining. **(C)** Representative images of MMP-9 expression in glioma cell lines by IHC staining. **(D)** Representative images showing the invasive capability of the glioma cell lines. **(E)** Comparison of GFAP expression levels by IHC staining scores. **(F)** Comparison of MMP-9 expression by IHC staining scores. **(G)** Comparison of invasive capability by cell numbers. Values represent the mean ± SD in triplicate, and the data represent one of three independent experiments. One-way ANOVA was used, ***p < 0.001.

After continuous subculture in vitro, the malignant degree of the glioma cell lines could become inconsistent with the pathological grade of their glioma tissues. To further identify the diversity of the malignant behaviors of cell lines in the two groups, we firstly detected the proliferation abilities of five cell lines and did not observe significant difference in proliferation abilities among the cell lines (Additional file 1: Figure S1). Then we conducted hematoxylin and eosin (HE) staining, immunohistochemical staining of GFAP and MMP-9, and a transwell assay to evaluate the differentiation level and invasion ability. The HE staining results showed three major shapes (circle, star and spindle) and obvious atypia of all these cell lines (Figure 3A). Immunohistochemical staining of GFAP was used to evaluate the differentiation level of the glioma cells. As shown in Figure 3B and 3E, cell lines of the high-grade group (U118, U87 and U251) had considerably lower GFAP positive expression compared with those of the low-grade group(CHG5 and SHG44), indicative of lower differentiation levels of cell lines of the high-grade group. Both the MMP-9 immunohistochemical staining and the transwell experiment were used to assess the invasion abilities of glioma cells. The results showed that the cell lines in the high-grade group possessed a generally higher invasion ability than those in the low-grade group (Figure 3C, 3D, 3F and 3G).

The comprehensive analyses of GFAP and MMP-9 expression levels, and invasion ability indicated that the cell lines from glioma tissues of different pathological grades showed obvious variations in malignant behavior. Compared with the low-grade group, the high-grade group showed a lower degree of differentiation and higher invasion ability. Thus, the two-cluster model of metabolic profiles was closely associated with the malignant behaviors of glioma cell lines cultured in vitro.

### Analysis of characteristic metabolites

**Figure 4:**
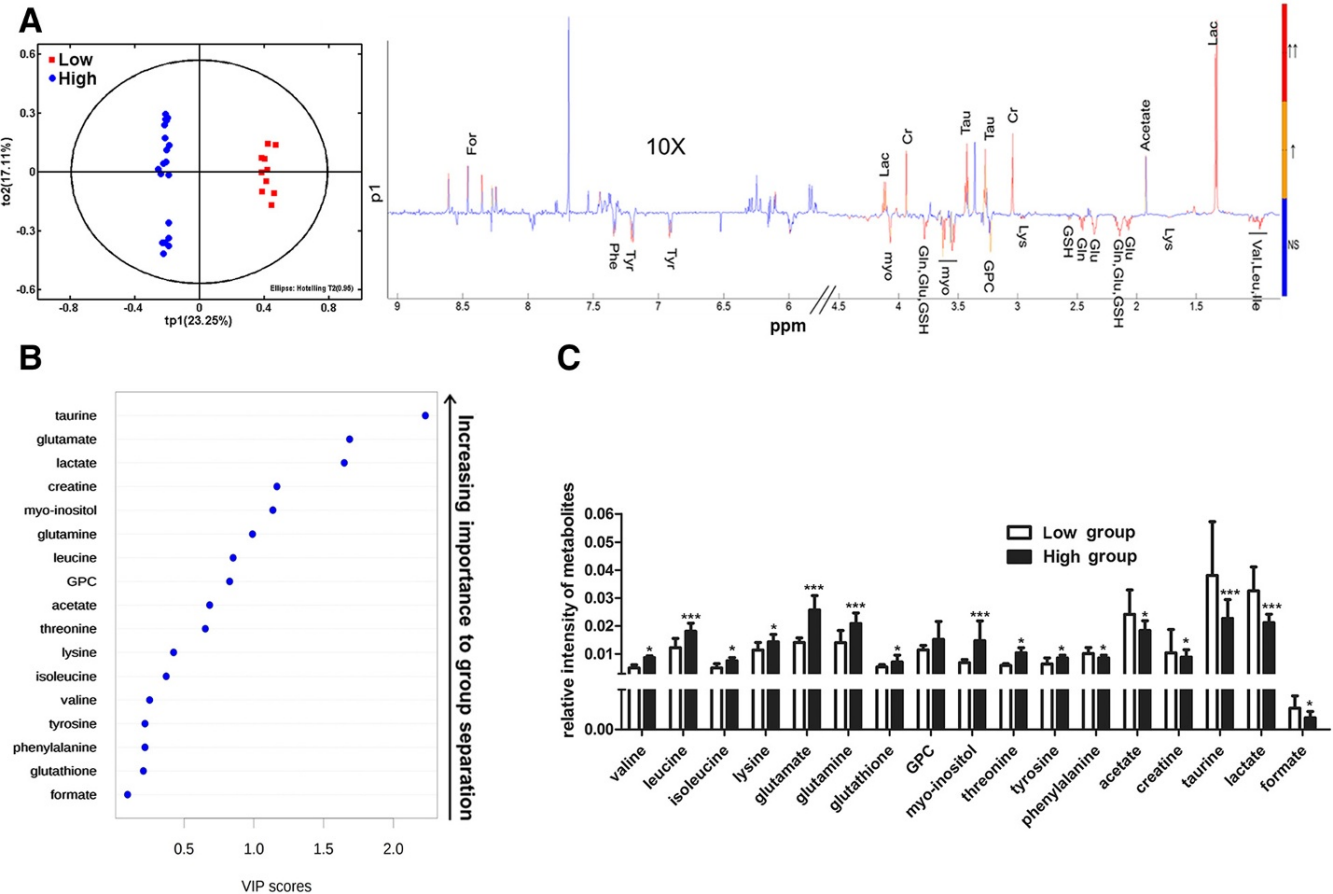
**Analysis of characteristic metabolites significantly responsible for distinguishing cell lines between the two groups. (A)** OPLS-DA score plot and OPLS-DA loading plot. The low-grade group includes CHG5 and SHG44; while the high-grade group includes U87,U251 and U118. The loading profile corresponds to tp1. The color of the loading plot can be used to identify the significant variables in the class separation. The red color indicates that the variables are very significant (|r| > 0.463 and VIP > 1); orange indicates that the variables are significant (0.362 < |r| < 0.463 and VIP > 1); and blue indicates that the variables are not significant (NS). **(B)** VIP rank-score of quantified metabolites. The overall VIP score from PLS-DA shown in the figure was the average across all the selected components. **(C)** The comparison of relative intensities of characteristic metabolites between the low-grade and the high-grade groups. (Student’s t-test was used, *p < 0.05; ***p < 0.001).

To identify the characteristic metabolites responsible for the separation between the low-grade group and the high-grade group, we applied OPLS-DA to analysis the difference in metabolic profiles of cell lines in the two groups. The scores and loading plots with correlation coefficients are shown in Figure 4A. The loadings plot was used to identify the significant characteristic metabolites responsible for the clustering patterns. Resonance assignments of these characteristic metabolites were according to literature references and the human metabolome database ( http://www.hmdb.ca). Approximately 17 characteristic metabolites were identified (Table 2), including: leucine (Leu), valine (Val), isoleucine (Ile), lysine (Lys), glutamate (Glu), glutamine(Gln), glutathione (GSH), threonine (Thr), tyrosine (Tyr), phenylalanine (Phe), taurine (Tau), sn-glycero-3-phosphocholine (GPC), myo-inositol (Myo), creatine (Cr), lactate (Lac), formate (For) and acetate (Ac).

The importance of the 17 metabolites in distinguishing metabolic profiles was ranked according to their VIP scores of the OPLS-DA model (Figure 4B). The results indicated that taurine had the highest correlation with the grouping of the glioma cells, followed by glutamine and lactate.

Additionally, we calculated the relative integrals of the characteristic metabolites for the two groups. Compared with the low-grade group, the high-grade group showed distinctly decreased levels of metabolites such as Phe, Cr, Tau, Lac, Ac and For, and significantly increased levels of other metabolites, including Val, Leu, Ile, Lys, Glu, Gln, GSH, GPC, Myo, Thr and Tyr (Figure 4C).

**Table 2:** **Characteristic metabolites from the OPLS-DA model of 1H NMR analysis of cell lines**

Metabolites (Abb)	Chemical shift (ppm)	Multiplicity	VIP
Valine	1.002	d	2.038
	1.053	d	
Leucine	0.966	t	2.970
	1.727	m	
Isoleucine	0.944	t	2.152
	1.021	d	
Lysine	1.734	m	1.108
	3.030	t	
Glutamate	2.055	m	2.573
	2.148	m	
	2.357	m	
Glutamine	2.144	m	4.110
	2.464	m	
Glutathione	2.177	m	1.870
	2.549	m	
	2.979	m	
Threonine	3.597	d	2.173
	4.258	m	
Tyrosine	6.915	d	1.377
	7.207	d	
Phenylalanine	7.341	m	1.294
	7.394	m	
	7.434	m	
Taurine	3.270	t	5.524
	3.426	t	
Glycerophosphocholine	3.235	s	3.983
	4.329	m	
Myo-inositol	3.534	d	3.410
	3.550	d	
	3.627	t	
	4.068	t	
Creatine	3.597	s	5.963
	4.258	s	
Lactate	1.337	d	9.514
	4.119	dd	
Formate	8.461	s	1.433
Acetate	1.920	s	5.618

Multiplicity: s singlet, d doublet, t triplet, dd doublet of doublets, m multiplet.

VIP: Variable importance in the projection was obtained from OPLS-DA model.

### Correlation analysis of characteristic metabolites

**Figure 5:**
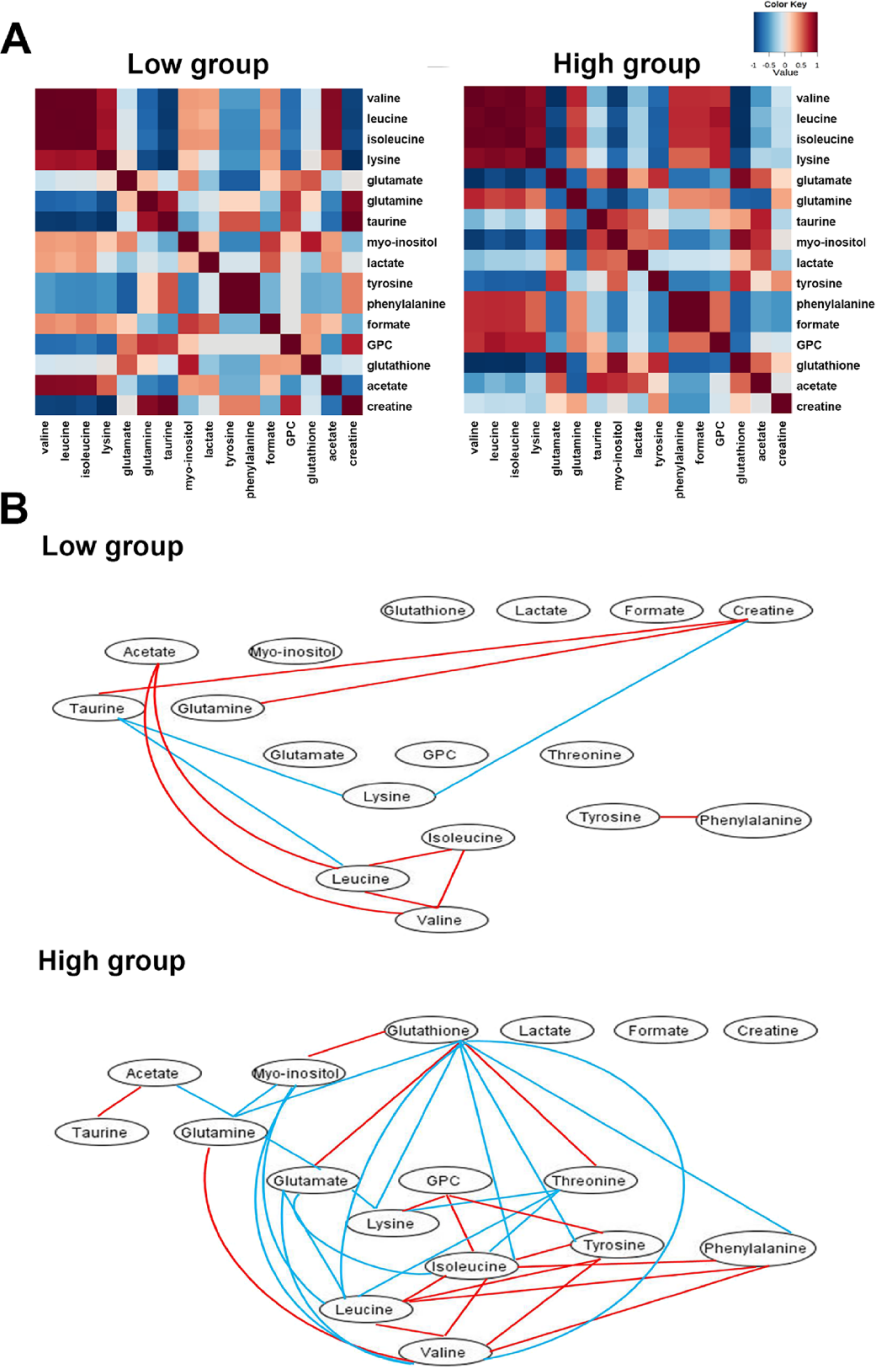
**Analysis of the correlation of the characteristic metabolites in the low-grade and high-grade groups. (A)** The heatmap of the Pearson’s correlation coefficients for characteristic metabolites in the low-grade and high-grade groups. The colors refer to the pair-wise correlation coefficients ranging from 1 (red) to -1 (blue). **(B)** Metabolic connectivity identified by the Pearson’s correlation coefficients in the low-grade and high-grade groups. The red color reflects a positive correlation, while the blue color reflects a negative correlation.

**Figure 6:**
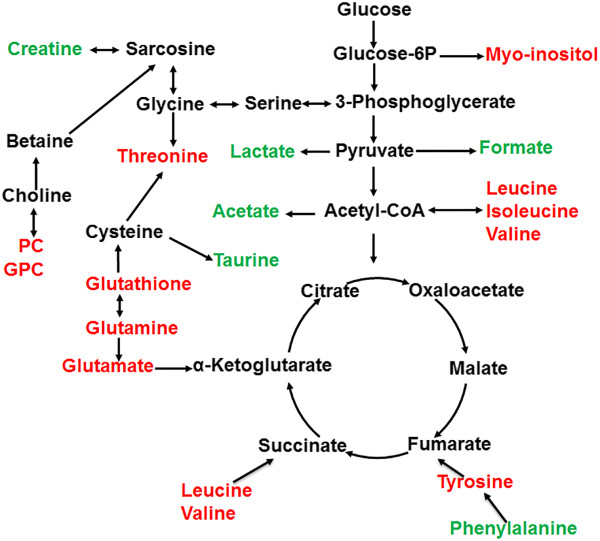
**Metabolic pathways of the malignancy-associated metabolites.** Compared with the low-grade group, the relative intensities of metabolites down-regulated in the high group are shown in green, whereas those up-regulated are shown in red.

To further clarify the potential relationships among the characteristic metabolites and the interrupted metabolic pathways in the two groups, we calculated Pearson’s correlation coefficients of the relative integrals of the characteristic metabolites (Figure 5A). Both the low-grade group and the high-grade group displayed quite different correlation patterns. Figure 5B show a network connecting the differently correlated metabolites in the two groups. Both groups displayed few similar correlations but many different correlations. Positive correlations of the branched chain amino acids (BCAA, including Ile, Val, Leu) in the low-grade group were also observed in the high-grade group. However, BCAAs in the high-grade group showed positive correlations with glutamine, tyrosine, phenylalanine, GPC; but negative correlation with myo-inositol, glutamate, threonine, glutathione, lactate, which disappeared in the low-grade group. Moreover, the positive correlations of BCAA with acetate observed in the low-grade group, disappeared in the high-grade group. Lysine was negatively correlated with taurine and creatine in the low-grade group, but disappeared in the high-grade group. Notably, the correlation patterns of the metabolites in the high-grade group were significantly different from those in the low group. Based on the different correlations of characteristic metabolites and the results from Kyoto encyclopedia of genes and genomes (KEGG) analysis, we summarized the potential disordered metabolic pathways that are associated with the malignant behaviors. As shown in Figure 6, the disordered metabolic pathways are mostly involved in TCA cycle anaplerotic flux, amino acid metabolism, anti-oxidant mechanism and choline metabolism.

In addition, we utilized the GeneGo pathway analysis platform to generate a protein-metabolite interactional network that could associate the characteristic metabolites with malignant behavior markers GFAP and MMP-9. The GeneGo analysis platform provides the prebuilt networks of protein-compound/metabolite assembled by GeneGo scientific annotators based on proven literature evidence [19]. Nine of the seventeen metabolites (valine, glutamine, glutathione, tyrosine, phenylalanine, leucine, acetate, choline, creatine) could directly connect to GFAP and MMP-9 pathway networks via the shortest path network option (Additional file 2: Figure S2). The altered levels of the nine metabolites were closely associated with the changed functions of GFAP and MMP-9, suggesting that these metabolites could be explored as potential biomarkers for monitoring malignant transformation of glioma cells.

## Discussion

Previous studies have documented the global metabolomic profiling of glioma tissues of different grades and considerable progress has been made toward understanding the underlying metabolic alterations associated with the progression of gliomas. However, researchers noted the limitation that ischemia and hypoxia cause metabolic degradation in glioma tissues, which could seriously affect the concentrations of specific metabolites. In the present work, we identified the malignancy-associated metabolomic signature directly from the glioma cell lines using ^1^H NMR-based metabolic profiling. According to references [1, 2], malignant gliomas(WHO II) and glioblastoma( WHO IV) are the most typical gliomas involved in malignant transformation. Thus, our study focused on glioma cell lines derived from glioblastoma (WHO IV) and WHO II grade glioma. To ensure the high reproducibility and accuracy of the metabolomic signature, six or more independent replicates of each cell line were obtained under the same conditions. The careful selection of cell cultures with the same growth conditions could ensure no significant differences among the metabolic profiles caused by the extracellular environment among the metabolic profiles.

According to the literature [20–22], we used the differentiation level and invasion ability to evaluate the malignant degrees of these cell lines after continuous subculture in vitro. The expression level of GFAP is an important index to assess the differentiation level of glioma cells [23]. In addition, previous studies have shown that MMP-9 plays an important role in the invasion process of glioma cells [24]. The malignant transformation of glioma cells is accompanied by increased expression of MMP-9 [25]. The measured GFAP and MMP-9 expression levels showed that the high-grade cell lines (U87/U118/U251) possessed a lower level of differentiation and a higher invasion ability than the low-grade cell lines (CHG5/SHG44), indicative of the consistency between the malignant degree of cell lines and the pathology grades of their glioma tissues after continuous subculture in vitro.

By the unsupervised and supervised analyses of ^1^H NMR data for the five glioma cell lines, we found that the metabolic profiles of cell lines CHG5 and SHG44 (WHO II)were separated from those of U87,U118 and U251(WHO IV). The clustering of metabolic profiles was consistent with the cell lines grouping based on the pathology grades of their glioma tissues. These findings indicated a close correlation between the metabolic profiles of cell lines and their malignant behaviors. Seventeen characteristic metabolites were identified to distinguish the metabolic profiles of the cell lines. The metabolic profiles from cell lines might provide information associated with their malignant features and other biological features; therefore, we further investigated the correlation of these characteristic metabolites with the glioma malignant behavior markers GFAP and MMP-9. Nine characteristic metabolites were directly connected to the GFAP and MMP-9 pathway network, including Val, Glu, GSH, Tyr, Phe, Leu, Ace, GPC and Cr. The levels of metabolites Val, Glu, GSH, Tyr, Leu and GPC significantly increased, while those of other three metabolites Ace, Cr and Phe decreased in the high-grade group. It seems that the altered levels of these metabolites could be associated with the differentiation and invasion process of glioma cells.

Notably, most of these altered metabolites in the two groups were amino acids. The altered amino acids in the tumor are mostly involved in TCA cycle anaplerosis [13] and protein biosynthesis [26]. It has been reported that Phe, Tyr, Leu and Val are involved in anaplerosis, which enter the TCA cycle by being converted into fumarate and succinyl CoA, respectively [27]. Moreover, glutamine is involved in anaplerosis through glutaminolysis, which enters the TCA cycle by being converted into glutamate initially and then into α-ketoglutarate [28]. Even though, the level of Phe was decreased, the amino acids including Val, Gln, Tyr, Leu were generally up- regulated in the high group, suggesting that more active TCA cycle anaplerotic flux might occur during malignant transformation of glioma cells.

In addition to increased amino acids, a higher level of GPC in the high-grade group was observed. As one of important choline-containing metabolites, GPC is involved in choline phospholipid metabolism of cell membranes. Alterations in choline phospholipid metabolism, with consequent alterations of these choline-containing metabolites, are a common feature of the cancer [29]. Thus, the increased level of GPC in the high-grade group might be related to the biosynthesis of cell membranes for rapid growth and high invasion ability. The metabolite GSH was also up-regulated in the high-grade group. As a ubiquitous intra- and extracellular protective antioxidant, GSH plays a key role in reducing reactive oxygen species and combating increased oxidative stress. A previous study reported that the level of reactive oxygen species in cancer cells was associated with malignant transformation [30]. In addition, GSH is particularly vital in protecting cells from radiation damage, which has generated reactive oxygen species. Rosi *et al.* found that a high level of GSH was correlated with radiation-induced apoptosis by MR spectra of cultured tumor cells [31]. Thus, consistently with previous literature [30, 31], the elevated GSH levels observed in our work might be related to enhanced antioxidant mechanisms in glioma cells with high malignant degree.

Decreased levels of creatine and acetate were observed in the high group. Both metabolites are usually detected in magnetic resonance imaging research of brain tumors. The creatine level reflect energy buffering and transport. Several studies have suggested that the creatine level in the low-grade gliomas is almost identical to that in the high-grade gliomas [29, 32]. Nonetheless, some studies observed that decreased creatine levels in brain tumors and in rectal cancer tissues at different stages [33, 34]. So far, the importance of creatine levels has remained unclear in the differentiation of low –grade from high-grade tumors [35]. The down-regulation of creatine observed in our work might support its biological importance in glioma grading. Acetate can either be transformed into acetyl-CoA, entering the TCA cycle, or be used as a precursor of membrane fatty acids [36], Nevertheless, the exact function of acetate in tumor cells remains unknown. Thus, the reason why acetate wa down-regulated in glioma cell lines in the high-grade group requires further investigations.

The other metabolites, including lactate, taurine, myo-inositol, lysine, isoleucine, threonine, formate, were not mapped into the GFAP/MMP-9 pathway network. However, some of them, such as lactate, taurine, myo-inositol, are metabolic markers frequently reported in cancers. Increased levels of lactate were detected in stomach cancer, oral cancer and rectal cancer tissues compared with the relevant normal tissues [34]. Interestingly, the decreased level of lactate was observed in cells of head and neck squamous cell carcinoma, compared with those of normal human oral keratinocytes. We also found a decreased lactate level in glioma cell lines in the high-grade group. Lactate production, due to the high glycolytic rates in cancer cells, enhances intracellular acidosis, which in turn leads to apoptosis. Previous works [37, 38] have also demonstrated that glioma cells could rapidly discharge lactate into the nearby microenvironment through monocarboxylate transporters. Thus, the lactate down-regulation observed in this work might be associated with the anti-apoptotic ability of glioma cells. Furthermore, taurine and myo-inositol are associated with osmo-regulation and volume regulation [13]. In our study, the levels of taurine and myo-inositol in the two groups were significantly different, implying that these metabolites might be involved in osmoregulation and volume regulation of glioma cells.

To the best of our knowledge, this is the first study to explore the malignancy-associated metabolic signature of on glioma cell lines with different malignant degrees using NMR-based metabolomic analysis. Consistent with other studies [14, 39], our results provide evidence that metabonomics analysis of cultured tumor cells is a valid method to understand the metabolic alterations accountable for their biological properties. Although we found that the characteristic metabolites are significantly associated with the malignant features of glioma cell lines, future works should be performed to further understand the regulation mechanism of the characteristic metabolites in malignant transformation.

## Conclusions

In the present work, we performed NMR-based metabolomic analysis on glioma cell lines with different malignant degrees. We have demonstrated that the metabolic profiles of the glioma cell lines are significantly associated with their malignant features. Moreover, we identified seventeen characteristic metabolites contributing significantly to distinguishing the metabolic profiles between the low-grade group and the high-grade group. These characteristic metabolites are primarily involved in dysregulation of metabolic pathways, including TCA cycle anaplerotic flux, amino acid metabolism, anti-oxidant mechanism and choline metabolism, which could correlate with the altered malignant behaviors of glioma cells. Our results lay the basis for both determining the molecular mechanisms underlying glioma malignant transformation and exploiting non-invasive biomarkers for the prognosis of glioma.

## Materials and methods

### Cell lines, cell culture and cell proliferation assay

Five astrocytoma cell lines from glioma tissues with different pathological grades (CHG5, SHG44, U87, U118, U251, Table 1) were used in the present work. Cell lines (CHG5 and SHG44) were kindly provided by Professor XW Bian of the Third Military Medical University, China. Glioblastoma U87 and U118 cell lines were obtained from the American Type Culture Collection (ATCC). Glioblastoma U251 cell line was obtained by the China Center for Typical Culture Collection (CCTCC). All the cell lines were maintained in DMEM supplemented with 100 units/ml penicillin, 100 μg/ml streptomycin and 10% fetal bovine serum (FBS, Hyclone) at 37°C in a humidified atmosphere of 5% CO_2_.

Five cell lines plated in 96-well plates (5 × 10^3^ per well) were cultured for 24 h and 48 h. Cell samples were then incubated with CellTiter 96 AQueous solution (MTS, 20 μl/well) and culture medium (DMEM, 100 μl/well) for 4 h. Next, colored MTS products were detected by absorbance at 490 nm on a Molecular Devices Microplate Reader (BioTek, USA).

### H&E and immunohistochemical stains

Cells were maintained on cover slips of 6-well plates in DMEM containing 10% fetal bovine serum as described above. At confluence, cells on cover slips were fixed with 4% paraformaldehyde for 15 min at room temperature, and were then stained with haematoxylin and eosin. Immunohistochemistry was performed as follows. Fixed cover slips were rinsed with 1% normal calf serum in PBS for 15 min, and permeabilized with 0.3% Triton X-100/PBS for 15 minutes. The cells were then incubated with primary antibodies specifically against GFAP(GA-5,MAIXIN-BiO) and MMP-9(56-1A4, MAIXIN-BiO) at 4°C overnight. The slides were washed with phosphate buffer solution (PBS) including 0.1% Triton X-100, incubated with biotinylated anti-mouse antibody (1:100) at 37°C for 1 hour, incubated with fluorescein isothiocyanate-labeled streptavidin conjugate (1:100) for 1 hour, and finally washed with PBS including 0.1% Triton X-100 three times, mounted, and analyzed under a microscope. The positive cells were counted and scored at a magnification of × 400 under a light microscope in five different fields for each coverslips. One-way analysis of variance (ANOVA) was used to determine the statistical significance of the differences among the five cell lines.

### Invasion assay

The invasion assay was performed using transwell cell culture chambers (24 wells, 8-μm pore size; BD Biosciences). 1 × 10^5^ tumor cells were resuspended in 200 μl of serum-free DMEM and added to the corresponding upper inserts, respectively. DMEM (600 μl) with 10% FBS was added to the lower chamber. After 24 h, invaded cells were fixed and stained with crystal violet (0.2% in 2% ethanol) for 20 min. Cells on the upper side of the insert membrane were removed with cotton rods. The invaded cells were counted at a magnification of × 100 under a light microscope in nine different fields for each insert. The measurements were repeated at least three times. One-way ANOVA was used to determine the statistical significance of the differences among five cell lines.

### Extraction of intracellular metabolites

Before metabolite extraction, 1 × 10^6^ cells were seeded in 10-cm diameter culture dishes and incubated for 48 h at 37°C and 5% CO_2_. About 5 × 10^6^ cells were then harvested and quenched by a direct cell quenching method, as described by Teng et al. [40]. Intracellular metabolites were extracted using a dual phase extraction procedure adopted from Viant et al. [41]. A mixture of methanol, chloroform and water in the volume ratio of 4:4:2.85 was used to generate a two-phase extract.

### NMR analyses of intracellular extracts

In the present study, only the aqueous intracellular extracts were used. Before NMR analysis, solvents were completely removed using a Nitrogen Blowing Concentrator. Each aqueous sample was reconstituted in 500 μl of D_2_O. Then 50 μl of D_2_O containing 1.5 M KH_2_PO_4_ and 0.1% sodium 3-(trimethylsilyl)propionate-2,2,3,3-d4 (TSP) was added. D_2_O was used for field frequency lock, and TSP was used to provide the chemical shift reference (d0.00). Subsequently, all the samples were vortexed, and centrifuged at 12000 g for 15 min at 4°C to remove any insoluble components. Finally the collected supernatants (500 μl) were transferred to 5 mm NMR tubes [42].

All ^1^H NMR experiments were conducted on a Bruker Avance III 600 MHz spectrometer at 25°C. Solvent-suppressed 1D NOESY spectra were acquired using the pulse sequence [(RD)-90°-t_1_-90°-τ_m_-90°-ACQ]. t_1_ was 6.6 μs. Water suppression was achieved by irradiation of the water resonance during the recycle delay ( RD ) of 4 s and the mixing time (τ_m_) of 120 ms. The spectral width was 10 kHz with an acquisition time per scan of 1.64 s, and 256 transients were collected into 32 K data points for each spectrum. The free induction decay (FID) was zero-filled to 64 K and an exponential line-broadening function of 0.3 Hz was applied to the FID before Fourier transformation. Both phase and baseline corrections were carefully performed. The ^1^H NMR spectra were referenced to the methyl group of TSP (δ 0.00).

### Multivariate statistical analysis

NMR spectra were reduced to 2587 integrated regions with a width of 0.003 ppm (bin) corresponding to the region of δ 9.5-0.8 using the MestRova6.5 software (Mestrelab Research S.L, Spain). The region of δ 5.5-4.5 was removed to eliminate artifacts related to the residual water resonance. (The remaining integrals for each NMR spectra were normalized to the sum of the spectral intensity to compensate for the differences in sample concentration) [43].

Before multivariate statistical analysis, the integral values were mean-centered and pareto-scaled [44]. To check general separation and identify the outliers, PCA was performed on NMR data sets of all cell samples using the SIMCA-P V12.0 software package (Umetrics AB, Umea, Sweden). Then, PLS-DA and OPLS-DA, were subsequently used to improve the separation [45]. The PLS-DA model was cross-validated to measure the robustness by a permutation analysis with 999 times.

FCM is a clustering method that allows one piece of data to belong to two or more clusters and is extensively used in pattern recognition [46, 47]. The FCM analysis was conducted by minimization of the following objective function:


Where m is any number greater than 1, u_ik_ is the degree of membership of x_i_ in the cluster k, x_i_ is the i-th measured data, v_k_ is the center data of the k cluster. We used the FCM clustering algorithm through the Fuzzy Logic Toolbox in MATLAB (Version MATLAB2011b, MathWorks, USA).

### Identification of characteristic metabolites and quantitative comparison

Two criteria were used to identify the characteristic metabolites. One was the VIP score of the OPLS-DA model [48] and the other one was the correlation coefficient(r) of the variable relative to the predictive component(t [1]) in the OPLS-DA model [49]. The critical values of correlation coefficients were determined by the degrees of freedom in the OPLS-DA model. Characteristic metabolites with a VIP > 1 and |r| > the critical values were identified.

For relative quantification of characteristic metabolites, the relative integrals of metabolites were used for comparison between two groups. The average changes and standard error were calculated [43].

### System statistical metabolic correlation and network analysis

Pearsons’s correlation coefficients of cell samples in two groups were further calculated to display the relationships between the relative integrals of spectral peaks in a certain biological profile, as described previously [43]. A heatmap was used to display the correlation matrices. For all correlations, a p-value was calculated based on a t-test to check the statistical significance. The significance threshold was set to the usual value of 0.05 and corrected according to the number of potential correlations [50].


The network of the metabolites with significance correlations was displayed by MATLAB Bioinformatics toolbox. Significant positive correlations were shown in red, while significant negative correlations were in blue. In addition, GeneGo MetaCore was used to analyze the network that describes the interaction between the characteristic metabolites and GFAP/MMP-9 [19].

## Electronic supplementary material

Additional file 1: Figure S1: The proliferation ability of five glioma cell lines. Differences in the 490 nm absorbance among five cell lines were compared by One-way ANOVA. Values represent the mean ± SD in triplicate. (TIFF 945 KB)

Additional file 2: Figure S2: Network of the shortest directional paths leading to characteristic metabolites related to GFAP/ MMP-9 regulation. Small molecules appear as purple hexagons, reactions as grey boxes, enzymes as orange shapes, transcription factors as red stars, ligands and extracellular peptides as green shapes, and transporters as purple "X" shapes. Metabolites with available data points are tagged with blue circles. Arrows denote mechanisms of interaction, in which green signifies activation and red indicates inhibition. Grey interactions represent transport or consumption/production of metabolic intermediates. (TIFF 3 MB)
