# Supplementary figures and images for: Malignancy-associated metabolic profiling of human glioma cell lines using 1H NMR spectroscopy
Source: Mol Cancer. 2014 Aug 27;13:197. doi: 10.1186/1476-4598-13-197 (PMC4158044; doi:10.1186/1476-4598-13-197)

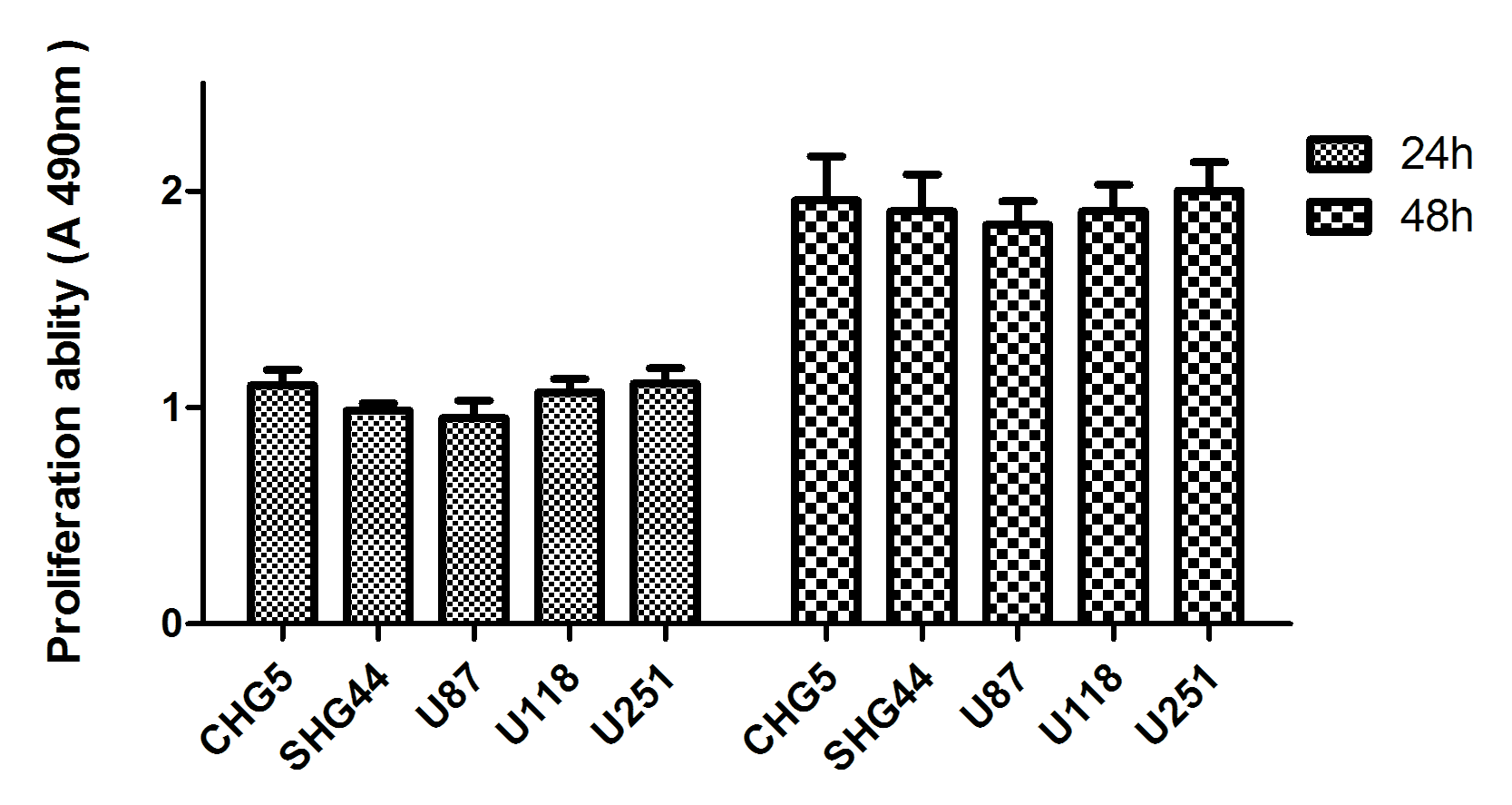

Supplement: Supplementary file 1 — Additional file 1: Figure S1: The proliferation ability of five glioma cell lines. Differences in the 490 nm absorbance among five cell lines were compared by One-way ANOVA. Values represent the mean ± SD in triplicate. (TIFF 945 KB) [file 12943_2014_1401_MOESM1_ESM.tiff]

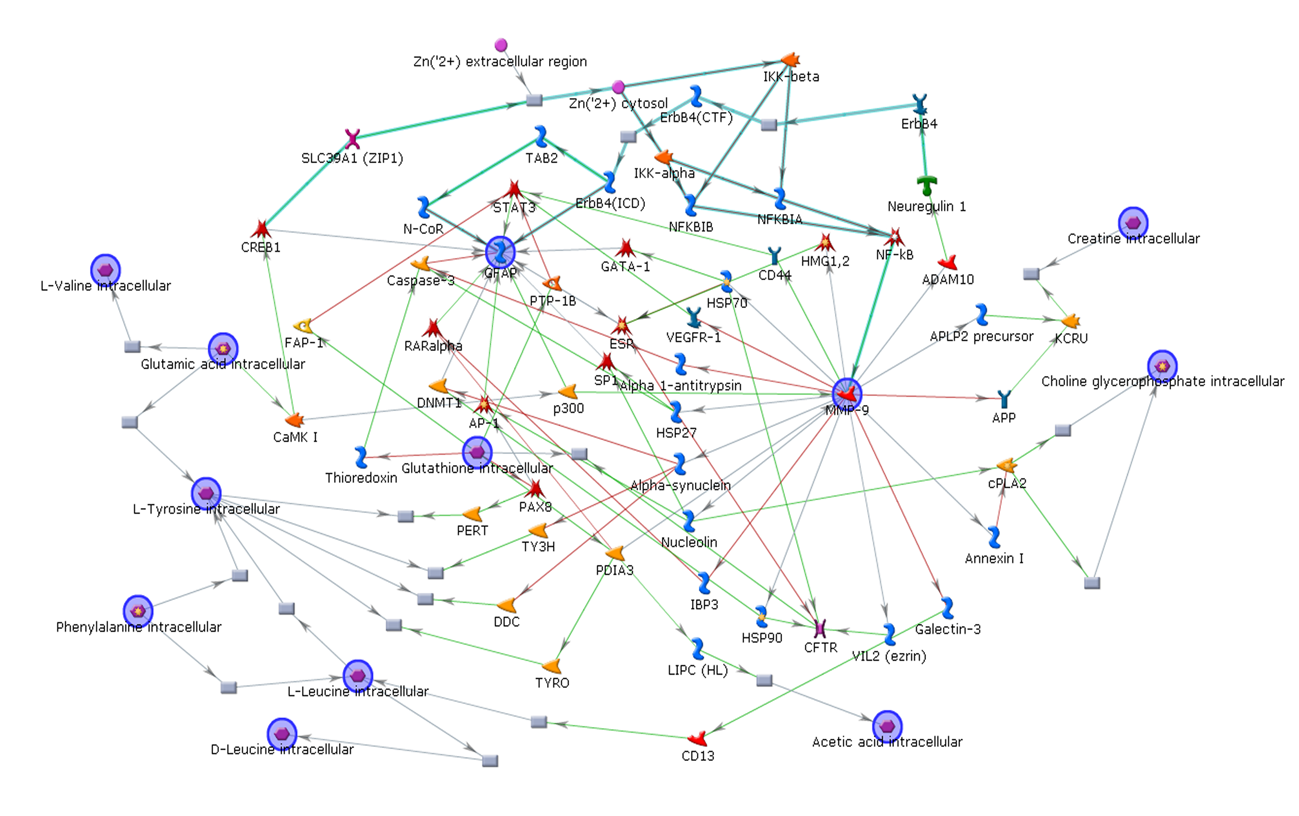

Supplement: Supplementary file 2 — Additional file 2: Figure S2: Network of the shortest directional paths leading to characteristic metabolites related to GFAP/ MMP-9 regulation. Small molecules appear as purple hexagons, reactions as grey boxes, enzymes as orange shapes, transcription factors as red stars, ligands and extracellular peptides as green shapes, and transporters as purple "X" shapes. Metabolites with available data points are tagged with blue circles. Arrows denote mechanisms of interaction, in which green signifies activation and red indicates inhibition. Grey interactions represent transport or consumption/production of metabolic intermediates. (TIFF 3 MB) [file 12943_2014_1401_MOESM2_ESM.tiff]
